# Non-Coding RNAs of Mitochondrial Origin: Roles in Cell Division and Implications in Cancer

**DOI:** 10.3390/ijms25137498

**Published:** 2024-07-08

**Authors:** Roberto Piergentili, Stefano Sechi

**Affiliations:** Istituto di Biologia e Patologia Molecolari del Consiglio Nazionale delle Ricerche, Dipartimento di Biologia e Biotecnologie, Università Sapienza di Roma, Piazzale Aldo Moro 5, 00185 Rome, Italy; roberto.piergentili@cnr.it

**Keywords:** mitochondria, non-coding RNA, cell division, cancer

## Abstract

Non-coding RNAs (ncRNAs) are a heterogeneous group, in terms of structure and sequence length, consisting of RNA molecules that do not code for proteins. These ncRNAs have a central role in the regulation of gene expression and are virtually involved in every process analyzed, ensuring cellular homeostasis. Although, over the years, much research has focused on the characterization of non-coding transcripts of nuclear origin, improved bioinformatic tools and next-generation sequencing (NGS) platforms have allowed the identification of hundreds of ncRNAs transcribed from the mitochondrial genome (mt-ncRNA), including long non-coding RNA (lncRNA), circular RNA (circRNA), and microRNA (miR). Mt-ncRNAs have been described in diverse cellular processes such as mitochondrial proteome homeostasis and retrograde signaling; however, the function of the majority of mt-ncRNAs remains unknown. This review focuses on a subgroup of human mt-ncRNAs whose dysfunction is associated with both failures in cell cycle regulation, leading to defects in cell growth, cell proliferation, and apoptosis, and the development of tumor hallmarks, such as cell migration and metastasis formation, thus contributing to carcinogenesis and tumor development. Here we provide an overview of the mt-ncRNAs/cancer relationship that could help the future development of new biomedical applications in the field of oncology.

## 1. Introduction

Mitochondria are maternally inherited cellular organelles present in most eukaryotic cells. Their first discovery dates back to 1856–1857, thanks to the studies of anatomist and physiologist Rudolf Albert von Kölliker, who identified “granules” in the sarcoplasm of striated muscle; they were then named “sarcosomes” by Retzius and “bioblasts” (life germs) by Richard Altmann in ca. 1890. Their present name was coined by Carl Brenda in 1898 from Greek thread, ‘mitos’, and granule, ‘chondros’ [1,2]. In 1899, Michaelis (of the Michaelis–Menten equation) discovered that the redox dye, Janus Green B (diethylsafranineazodimethylaniline), served as a specific supravital stain for Altmann’s bioblasts [3]; yet, curiously, he did not relate this finding to a possible role of mitochondria in cellular oxidations. Only in 1953 did Lazarow and Cooperstein show that this staining is indeed due to mitochondria’s capacity to reoxidize the reduced dye by way of cytochrome oxidase [4]. Imaging with Janus Green B remained the key marker of these structures until the first high-resolution electron micrographs published in 1952 by Palade [5]. In 1914, Warburg, winner of the Nobel Prize in Physiology or Medicine in 1931 for his work on respiratory chain enzymes, identified mitochondria as the site of cellular respiration [6]. The appellation “powerhouse of the cell”, was coined by Philip Siekevitz in a popular article published in 1957 [7]. Indeed, it is now well known that mitochondria are the main site of oxidative respiration, and thanks to their action, resources such as glucose are used to form energy supplies, e.g., ATP and NADH, that can be readily used by the cell, producing—considering together glycolysis, the citric acid cycle, and the electron transport chain—38 molecules of ATP for each catabolized molecule of glucose.

## 2. The Mitochondrion

### 2.1. Role of Mitochondria in Human Health

Since mitochondria are responsible for the production of ca. 90% of the cellular energy [8], they are involved in several metabolic pathways including tricarboxylic acid cycle (TCA), β-oxidation of fatty acids, calcium metabolism, and ROS (reactive oxygen species) detoxification [9,10]. They take part in the control of intrinsic apoptosis, autophagy, and senescence [11], and their dysfunction is strongly connected to several human pathologies. The first human mitochondrial DNA (mtDNA) mutations were described in 1988 and involve muscular and neuronal systems, which is not surprising considering the high demand of energy in these tissues [12,13,14]. However, additional mitochondria-related conditions had been identified in the years, such as diabetes and deafness [15,16], Parkinson’s disease (PD) [17], Alzheimer’s disease (AD) [18], seizure activity and neuronal death [19,20], non-alcoholic fatty liver disease (NAFLD) [21], liver oxidative stress [22], adaptation to hypoxia [23], iron [24] and calcium [25] homeostasis, and cancer, where mtDNA mutations drive the rewiring of bioenergetics and biosynthesis [26]. In addition to specific pathological states, mitochondria functions are also closely linked to another physiological process, beyond energy production: cell senescence, i.e., the irreversible loss of cellular replicative potential [27] strictly connected to age-related chronic pathologies. In aging cells and tissues, the oxidative capacity of mitochondria declines over time, together with a decreased mitochondrial membrane potential (MMP) at steady state, although mitochondria accumulate in those cells, likely because of impaired mitophagy [28]. This dysfunction causes diminished energy production, altered NAD+/NADH balance [29] and intracellular accumulation of ROS, able to cause persistent DNA damage, in turn, linked to mutation and cancer formation [30,31]. Indeed, in many instances a direct link has been shown between aging and the dysfunction of various OXPHOS (oxidative phosphorylation) complexes (reviewed in [27,32,33,34]).

### 2.2. Discovery and Characterization of Mitochondrial DNA

**Figure 1:**
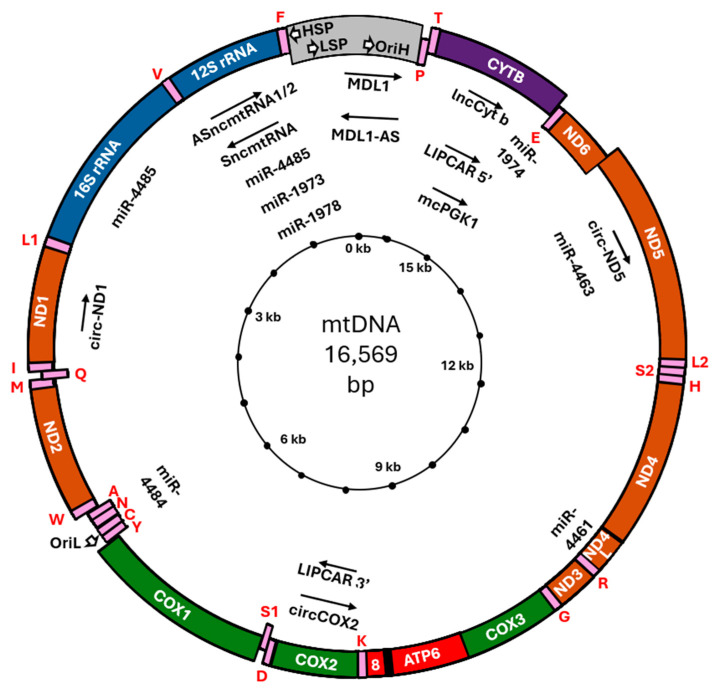
Schematic representation of mtDNA content. From outer to inner layers: schemes of H strand (outer) and L strand (inner), respectively, containing 2 rRNA genes, 22 tRNA genes, and 13 protein coding genes; ncRNA approximate map position; nucleotide position map with total mtDNA length and 1 kb subdivisions (each dot represents counterclockwise 1 kb subdivisions starting from zero, on top). Genes are staggered according to the strand (H/L) they map and are identified as follows. Grey box: control region (D-loop), containing HSP and LSP (transcription promoter of H- and L-strand, respectively) and the origin of replication for the heavy strand (OriH); note that OriL (origin of replication for the light strand) is outside this box and maps between N and C tRNA genes (bottom left). Blue boxes: ribosomal RNA (rRNA) genes. Pink boxes: transfer RNA (tRNA) coding genes; each gene is indicated with a single letter code (red) corresponding to the amino acid encoded in its anticodon; S1/2 and L1/2 represent codon usage variants. Brown boxes: genes encoding NADH dehydrogenase subunits (ND1-7). Green boxes: genes encoding cytochrome c oxidase subunits (COX1-3). Red boxes: genes encoding ATPase subunits (ATP6 and 8). Purple box: gene encoding cytochrome b (CYTB). Black boxes: overlap of genes ATP6/8 and ND4/4L. In the ncRNA map, arrows indicate long ncRNA transcription direction; the position of miRs, due to their length, is approximate. See text for further explanations and references.

The first hypothesis that mitochondria might “bear genes” is due to Friedrick Meves [35] and Claudius Regaud [36], although at those times the concept of “gene” and their cellular localization was far from fully elucidated. In 1963, for the first time, thread-like structures were identified inside chicken embryo mitochondria, that could be digested by DNase, but not by RNase, showing the presence of DNA inside these structures [37]; similar results were also soon obtained in yeast [38] and *Neurospora* [39]. In the following years, it was also demonstrated that mtDNA is species specific, circular, ~5 μM long, transcriptionally active, and contains genes different from those harbored by the nucleus (nuclear DNA, or nDNA) (reviewed in [2,40]). The double DNA helix is identified as sense strand and antisense strand, and the hemihelices are named as heavy (H) strand and light (L) strand, respectively. In human cells, mtDNA is 16,569 base pairs long and contains 37 genes, including 13 polypeptide-encoding genes, two ribosomal RNAs (rRNA) genes, and 22 transfer RNAs (tRNA) genes (Figure 1). The 13 polypeptides embody the core subunit of the OXPHOS complexes I, III, IV, and V, and are essential for its activity [41], although they represent a minority of the ~100 proteins involved in OXPHOS function. In addition, there is one non-coding control region (NCR; also called D-loop region) harboring regulatory elements including the displacement loop (D-loop), which is essential for mtDNA replication and transcription [42]. Mitochondrial DNA also harbors a growing list of short open reading frames (sORFs) that encode mitochondrial-derived peptides with systemic functions, such as humanin (HN), which protect neurons from death caused by several early-onset familial AD genes [43], PD, cardiovascular diseases, inflammation, diabetes, obesity [44], and MOTS-c, which activates antioxidant response [45] and regulates insulin sensitivity [46].

## 3. Mitochondria and Cancer

A tumor cell is essentially a cell that has lost the ability to regulate its own replication cycle and, consequently, it is characterized by a fast proliferation rate. In solid tumors, proliferating cells rapidly form a mass that shapes—and it is shaped by—the tumor cells themselves. In particular, the mass is characterized by the different metabolic needs in respect to its surrounding, healthy tissues. Rapid growth is sustained by the afflux of oxygen and nutrients, and in this environment the tumoral mass rapidly gains the ability to promote the growth of blood vessels (angiogenesis) to fulfill these needs. Yet, the newly formed vessels are far from being comparable to their normal counterparts: the architecture of the tumor vasculature is leaky, tortuous, fragile, and blind-ended [47]. Another problem faced by tumor cells is the presence, especially in the inner layers of the mass, of low oxygen saturation regions, causing hypoxia, which challenges the oxidative metabolism to supply energy for the rapid growth and induces a gene expression program switch that allows tumor cells to adapt to hypoxic stress. Indeed, it is well known that tumoral cells drastically change their catabolism, displaying a decreased respiration and an increased glycolysis, proportional to the increase in their growth rate [48,49]; this is also known as the “Warburg effect”, named after its first discoverer, Otto Warburg [50,51]. Studies show that hypoxic stress is a frequent event during tumorigenesis and adaptation of tumor cells to low oxygen concentration promotes, among the others, the selection of drug-resistant and invasive phenotypes [52,53]. These adaptative changes, as well as neoplastic transformation per se, are mainly driven by mutations in nDNA, but several mtDNA mutations have also been characterized, linking mitochondrial genome alterations to tumorigenesis and tumor development. There are two main classes of mtDNA alterations in cancer: mutations that promote cancer formation/development (inducers) and mutations that allow cells to adapt to the changing tumor microenvironment (adaptors) [54]. Similarly to their nDNA counterparts, mtDNA changes may be maternally inherited variants, de novo somatic mutations, and ancient adaptive polymorphisms (haplogroups); copy number variations (CNV) of mtDNA have a role in cancer biology as well [54]. For example, cytochrome oxidase subunit I (COI) gene mutations in highly conserved amino acids affect 11–12% of all patients with prostate cancer [55]. In the same tumor, Kalsbeek and collaborators identified 74 unique prostate cancer-specific somatic mtDNA variants in 50 patients showing a significant positive correlation among the total burden of acquired mtDNA variation, elevated Gleason score at diagnosis and biochemical relapse [56]. Hopkins et al. analyzed 384 localized prostate cancer samples and found co-occurrence between specific mtDNA and nDNA pathogenic mutations. For instance, certain control region mtSNVs (mitochondrial somatic mutations) co-occur with the gain of the MYC oncogene, and these mutations are jointly associated with patient survival [57]. The same group found similar results in pancreatic ductal adenocarcinoma, again showing co-occurrence of pathogenic mutations in mtDNA and nDNA [58]. Beyond prostate cancer, one of the best characterized tumors in respect to mtDNA mutations, other tumors had been studied as well. Ju and collaborators analyzed somatic alterations in mtDNA from 1675 tumors from over thirty different types of cancer and identified 1907 somatic substitutions, mostly being C > T and A > G transitions localized on the mtDNA heavy strand [59]. Interestingly, though, this work did not highlight any direct advantage brought by these mutations to cancer growth/diffusion, suggesting that mutations strongly impairing mitochondria function are negatively selected—tumor cells still need mitochondria, although they likely fulfill different roles when compared to normal cells. However, the ratio of missense–silent substitutions was 4.2 (878 vs. 208, respectively), suggesting a selective bias towards the acquisition of functional mutations in tumors, possibly related to the lower oxidative metabolism of these organelles inside cancer masses. In line with the previous report, Grandhi and collaborators [60] analyzed whole-genome sequence data from 1916 patients across 24 cancer types to characterize patterns of mtDNA mutations, finding that tumor mitochondrial genomes show distinct mutational patterns and are disproportionately enriched for protein-altering changes; in addition, these mutations—rare in normal cells—preferentially expand in the altered tumor environment, suggesting selective advantage. This is particularly evident in chromophobe renal cell carcinoma and thyroid cancer, especially for truncating mutations. Comparable results on truncating mutations were found by Yuan and collaborators, who analyzed whole-genome sequencing data from 2658 cancers across 38 tumor types [61]. They found that truncating mutations are markedly enriched in kidney, colorectal, and thyroid cancers, suggesting oncogenic effects with the activation of specific signaling pathways. They also found markedly mtDNA CNV in specific tumors, such as ovarian cancer, kidney chromophobe, chronic lymphocytic leukemia, lung squamous cell carcinoma, pancreatic adenocarcinoma (increased mtDNA copies), kidney clear cell carcinoma, hepatocellular carcinoma, and myeloproliferative neoplasm (decreased mtDNA copies). An analysis of mtDNA CNV was performed on 22 tumor types profiled by The Cancer Genome Atlas (TCGA) project also by Reznik et al. [62], who observed a tendency for some cancers, especially of the bladder, breast, and kidney, to be depleted of mtDNA, compared to adjacent normal tissue. In these tumors, there is a concomitant reduction of the expression of mitochondrial genes, suggesting a suppression of mitochondrial activity in these tumor types. Hundreds of published works exist linking single mtDNA mutations to specific cancers; for these, we redirect readers to comprehensive reviews of the literature [54,63,64,65].

An interesting discovery regards numtogenesis [66,67], i.e., the transfer of mtDNA sequences to the nDNA, a potentially new route to link mtDNA and human cancer. Numtogenesis had been identified in 1991 in HeLaTG cells [68]. This transfer may involve small fragments of mtDNA or the entire mitogenome. In fact, it has recently been demonstrated, through targeted experiments on healthy and maternally related individuals, the presence of nuclear elements of mtDNA (NUMTs), which represent complete mitogenomic sequences in multi-copy format (Mega-NUMTs) [69,70]. To date, more than 1000 insertions of NUMTs have been identified in the nuclear genome. The process of integration of NUMTs into the nuclear genome is of particular interest because the insertion of these elements can occur within a gene, thus destroying and promoting both rare and more common disease processes, such as cancer [71]. Indeed, numtogenesis has been linked to malignant cervical epithelial cells [72], colorectal adenocarcinoma [73], breast cancer [74], lung, skin, breast, and uterine cancers [61], although additional studies are required to verify the actual mechanism and role of this phenomenon in tumorigenesis [66,67].

The crosstalk between nDNA and mtDNA further extends to the activity of ribosomes. In fact, the mitochondrial ribosome (called mitoribosome) biogenesis includes correct maturation and folding of the mitochondria-encoded RNA components (12S and 16S mt-rRNAs, and mt-tRNAVal) and their assembly together with 82 nucleus-encoded mitoribosomal proteins (reviewed in [75,76]). Ribosome biogenesis is also strongly altered during neoplastic transformation; in these cells is possible to observe an increase in ribosome production associated with aberrant ribosome biogenesis homeostasis and alteration in number, size and shape of nucleoli, together with the alteration in rRNA synthesis and the deregulation of some mitochondrial or cytosolic ribosomal proteins; this causes a condition known as nucleolar (or ribosomal) stress which, in turn, causes the translocation to the nucleoplasm of specific cytosolic ribosomal proteins that there exert non-ribosomal functions (reviewed in [77]). For example, it has been shown in mice an involvement of p53 during nucleolar stress [78]; in man, several kinds of cancer mutated of retinoblastoma protein (RB) and p53 show the upregulation of rRNA synthesis coupled with a more aggressive phenotype compared with similar tumors not mutated in those genes [79] and also c-Myc is a recognized player in this scenario [80]. Additional tumor-related cellular processes involving nucleolar stress include cell cycle control, DNA repair, maintenance of genome integrity, cellular proliferation, apoptosis, autophagy, cell migration, and invasion [77,78,81].

We conclude this section by recalling that, at present, the role of mitochondria in cancer genesis, development, and spread is not fully elucidated. On the one hand, there is established evidence of the lower oxidative metabolism of tumor masses; yet, it has been also repeatedly shown that completely impairing mitochondrial function in cancer cells causes their death [82]. Thus, clearly, mitochondria are still important in cancer cell metabolism, though they might fulfill a different role, in respect to normal cells. Recently, an innovative working hypothesis has been presented, aiming at explaining the apparent paradox between high energy demand and (relatively) low energy production in cancer cells (lower oxidative mitochondrial function and enhanced glycolysis) and the concomitant need of fast growth and metabolites accumulation [83]. According to this hypothesis, “enhanced glycolysis partially inactivates oxidative phosphorylation to induce functional rewiring of a set of TCA cycle enzymes to generate new non-canonical metabolic pathways that sustain faster growth rates”. In this scenario, rewired mitochondria would receive products derived from glycolysis and transform them into metabolites that, combined with those coming from glycolysis, might generate all the molecules (nucleotides, proteins, and fatty acids) necessary to produce a new biomass, thus allowing faster anabolic reactions if compared to reactions guided by wild type mitochondria. Additional studies are required to either validate or confute this hypothesis.

## 4. Non-Coding RNA and Cancer

The human genome contains approximately 20,000 protein coding genes [84,85], which represent ~1–2% of the total DNA contained within a cell. However, the portion of human genome that is transcriptionally active is estimated at around 93%, meaning that the majority of these transcripts comes from intergenic sequences; globally, they exceed 120,000 non-coding transcriptional units [86,87]. Though, the exact number of transcripts with a biologically relevant role is currently unknown [88]. These non-coding RNAs (ncRNA) are broadly divided into two main classes, according to their length: long ncRNA (lncRNA, exceeding 200 nucleotides, nt) and short (or small) ncRNA (sncRNA, shorter than 200 nt and mostly in the range of 20–30 nt). Although most of the mass (>95%) of ncRNA inside a cell is represented by rRNA and tRNA [88], the remaining ncRNAs play pivotal roles in several aspects of cell life, including cell replication. Consequently, increasing evidence is accumulating linking ncRNA deregulation and cancer. Long ncRNA are a class of molecules that are heterogeneous in several aspects; they have structural differences (there are linear and circular RNA), different genomic locations (intergenic, intronic, sense and antisense transcripts) and, above all, diverse functions [89,90]. In some instances, lncRNA act as a sponge for a specific class of sncRNA, called microRNA (miRNA or miR), impairing their inhibitory action on target mRNA; in these cases, lncRNA are referred to as “competing endogenous RNA” (ceRNA) and the mRNA-miR-lncRNA interaction is described as an axis inside a more complex network of interactions, collectively called ceRNA network (ceRNET). These networks had been described in several cancers, such as leukemia [91], glioma [92] and breast cancer [93]. In other cases, lncRNA may be the scaffold on which two or more proteins, or even another RNA, load to create functional complexes or are brought together to interact with each other, either to enhance or inhibit their function [94,95]. Some lncRNA are antisense (AS) transcripts, i.e., they are transcribed from the opposite strand of genes with either protein-coding or non-coding function and may act both as stand-alone lncRNA performing independent functions and/or by directly binding their complementary sequence and modulating its expression [96]. Interestingly, some lncRNAs (including AS-RNA) are also able to induce chromatin conformational changes, thus potentially regulating numerous genes at the same time [97]. In all cases, lncRNA action in neoplastic transformation may be either oncogenic or oncosuppressive, depending on their targets, cancer type, or specific function.

Interestingly, ribosome biogenesis in health and disease is influenced by nDNA-derived lncRNA activity. As also reported in the previous section, in tumor cells, increased and synchronized mitochondrial and nuclear ribosomal biogenesis are observed, both are necessary to sustain the needs of the tumor cell such as enhanced growth and faster cell division [77]. To date, the literature lacks examples of mitochondrial ncRNA affecting nuclear ribosome biogenesis. However, there are examples in the opposite direction. In fact, the role of SAMMSON (survival-associated mitochondrial melanoma-specific oncogenic non-coding RNA) is well documented. SAMMSON is a long non-coding RNA of nuclear origin that is directly involved in both nucleolar and mitochondrial synthesis of ribosomes. It interacts with p32 and XRN2 proteins, increasing their localization in mitochondria and nucleus, respectively. P32 is a protein essential for mitoribosome formation [98], while XRN2 is an exonuclease necessary for pre-rRNA processing [99]. SAMMSON is described as a pro-oncogene involved in mitochondrial homeostasis and metabolism that is expressed in aggressive melanomas, where it promotes cell growth, while its downregulation in melanoma xenografts suppressed the tumor growth, making it an important potential therapeutic target in melanoma patients [100,101].

MicroRNAs are also important in carcinogenesis. They belong to the sncRNA class and exert their function by directly binding homologous sequences on target mRNAs (mostly in the 3′ UTR), either promoting mRNA degradation or impairing its translation. As such, their main role is suppressing gene expression [102]. Due to their limited length and to the ability to bind targets even in presence of minor sequence mismatch, each miR can bind several mRNA targets [103]. The human genome supposedly harbors more than 2500 miR, which regulate over 60% of human genes [104]. When mRNA targets include known oncogenes or oncosuppressors, miR are directly involved in tumor formation and development; indeed, miR’s role in cancer is a well-established fact [105,106] and their action in this pathology is either direct (mRNA targeting) or indirect (ceRNET, described above). In the literature, there are many works that describe the presence of miRNAs within mitochondria (reviewed in [107,108,109,110,111]); however, the nuclear or mitochondrial origin of these miRNAs is elusive. In fact, aspects such as the identification, role, and origin of miRNAs are often based on results obtained using combinations of bioinformatic tools or through the comparison of the sequence alignment between the miRNA and the mitochondrial genome [112,113,114]. This approach has limitations since the miRNAs that are derived from transcripts of numerous nuclear pseudogenes of mitochondrial DNA origin (the above-mentioned numtogenesis) can be mistakenly confused with mitochondrial transcripts and this error is inversely proportional to the length of the nucleotide sequence examined [115,116,117]. Some groups have hypothesized that the mitochondrial genome may encode miRNAs because, within the mitochondrion, they identified proteins involved in their biogenesis, such as Dicer [118,119] or AGO2 [113,118,120,121,122,123]. However, these data were not validated by other groups using the same system. Furthermore, to date, there is no experimental evidence of the presence within the mitochondrion of the Drosha and DGCR8 proteins, which play a key role in miRNAs biogenesis [124]. Actually, miRNAs biogenesis, beyond the canonical pathway which includes Drosha, DGCR8 and Dicer proteins, also occurs through non-canonical pathways [125,126,127,128]. Furthermore, the mitochondrion has its own transcriptional apparatus [129], thus it is plausible to speculate that the potential mitochondrial transcription of miRNAs could occur using molecular systems that are not yet characterized or that are partially different from those used for the biogenesis of nuclear miRNAs. The work of Ro et al. in 2013 has been among the first to support the hypothesis that the mitochondrial genome can indeed transcribe miRNAs [130]. In this work, the authors performed a deep sequencing analysis to compare the transcriptome between Rho0 (mtDNA-depleted)—PNT1A (human prostate epithelial cell line) cells and wild-type cells. The results show a general decrease of miRNAs in Rho0–PNT1A cells, compared to the control, which is correlated with the depletion of mitochondrial DNA, suggesting that some of those miRNAs might indeed be transcribed directly from the mitochondrial genome.

In the last few years, evidence has accumulated showing that mtDNA encodes ncRNA, both long and short, beyond the rRNA coding gene (Figure 1). These RNAs fulfill several roles in cell metabolism and homeostasis and indeed, their dysfunction is currently being investigated in many laboratories as they are implicated in numerous human conditions, including (but not limited to) cancer. We report below the most up-to-date information about mtDNA-encoded ncRNA and their function in the control of tumor cell growth, metabolism, invasion, and angiogenesis.

## 5. Mitochondrial lncRNA

### 5.1. SncmtRNA, ASncmtRNA-1, ASncmtRNA-2, miR-4485 and miR-1973

The first pioneering works performed in mouse cells demonstrated that their mitochondrial genome encodes a chimeric ncRNA formed by the sequence of the mitochondrial 16S rRNA, contained in the H filament, plus 121 transcribed from the L filament of the same gene that forms an inverted repeat (IR) [131,132]. In situ hybridization (ISH) analysis using a probe complementary to the 16S rRNA shows that the ncRNA is overexpressed in mouse and human proliferating spermatogonia, suggesting a correlation with cell replication [133]. In 2007, Villegas and collaborators identified in human cells ncmtRNA, an RNA molecule coded by the sense strand (recently re-named SncmtRNA) structurally resembling that of the mouse, which represents the first lncRNA encoded by human mtDNA [134]. Human SncmtRNA is a transcript of 2374 nt that forms a hairpin structure that includes both an 815 nt IR, generating a perfect double-stranded stem, and a 40 nt loop. ISH analysis using probes specific for SncmtRNA allowed showing that this transcript is overexpressed only in proliferating tumoral cell lines, such as HeLa (human uterine cervical carcinoma cell line), SiHa (human cervical squamous cell carcinoma cell line), DU145 (prostate cancer cells), and MCF7 (human breast cancer cell line expressing estrogen, progesterone, and glucocorticoid receptors), but not in non-proliferating control cells (human brain, smooth muscle, and liver cells) [134].

This finding was later confirmed using additional proliferating tumor cell lines, such as MDA-MB-231 (breast carcinoma), OVCAR3 (ovary carcinoma), and Jurkat (acute T cell leukemia) cells [135]. Interestingly, though, the authors noticed that the transcript is also present in normal proliferating cells, such as human umbilical vein endothelial cells (HUVEC), human foreskin keratinocytes (HFK), and human tonsil endothelial cells (HUTEC) [135]. The observation that this lncRNA is expressed in proliferating cells, either tumoral or healthy, and not in non-proliferating cells, suggests a general role for SncmtRNA in cell division. Two additional experimental observations on lymphocytes treated with phytohemagglutinin (PHA) and DU145 cells treated with aphidicolin, strengthen this hypothesis. Human circulating lymphocytes do not express SncmtRNA but PHA treatment stimulates their entry into the S phase, as demonstrated both by DNA synthesis measured by BrdU incorporation and by the expression of the proliferative antigen markers Ki-67, PCNA, and phosphohistone H3. Conversely, in proliferating DU145 cells expressing high levels of SncmtRNA, aphidicolin-mediated arrest at G1 phase induces the downregulation of SncmtRNA transcription. Removal of the drug reverses the cellular phenotype, returning SncmtRNA transcription to levels similar to untreated cells [134].

Using HUVEC-, HFK-, and PHA-stimulated lymphocytes, two 16S rRNA antisense transcripts called ASncmtRNA-1 and ASncmtRNA-2 were identified. The structure of the two antisense transcripts is similar to that of SncmtRNA. In fact, both ASncmtRNA-1 and ASncmtRNA-2 contain a hairpin structure that includes an IR and a loop of 310 nt and 96 nt, respectively, for ASncmtRNA-1, and of 545 nt and 450 nt, respectively, for ASncmtRNA-2. These two antisense transcripts are expressed in normal human proliferating cells but are downregulated both in tumor cell lines and in tumor cells isolated from biopsies of 17 different tumor types [135].

Analysis of the cellular localization of SncmtRNA and ASncmtRNA on both normal and tumor human and murine cells and tissues suggests their role in retrograde mitochondrial–nuclear signaling. Using different experimental approaches, such as ISH, confocal microscopy, in situ digestion with RNase before ISH and electron microscopy ISH, the cellular localization of both SncmtRNA and ASncmtRNA was analyzed. The results obtained from the analysis of normal human tissues (pancreas, spleen, and kidney) and of human biopsies of lymphoma, cervix, and renal carcinoma show the nuclear localization of SncmtRNA. Instead, ASncmtRNA localizes to the nucleus in healthy tissues but, as previously described, in tumor tissues it is downregulated [135]. Furthermore, even in normal cells, such as human kidney and mouse testis, and in tumor cells (human renal cell carcinoma and mouse melanoma cells), SncmtRNA appears to be associated with nucleoli and heterochromatin [136]. Both ASncmtRNAs share the same localization as SncmtRNA only in normal tissues. However, the authors specify that the nuclear localization of SncmtRNA in tumor cells is preferential and not universal. Indeed, ISH performed on biopsies of breast, prostate, lung, cervical and thyroid tumors revealed that SncmtRNA shows a different localization, either nuclear or cytoplasmic, when comparing biopsies of the same tumor type obtained from different patients [136]. Although further studies are needed to clarify the significance of this different distribution, a plausible explanation could be the involvement of these lncRNAs in epigenetic regulation related to the early stages of malignant transformation.

The observation that SncmtRNA is expressed in all kinds of proliferating cells (both normal and tumoral), while ASncmtRNAs is expressed only in normal proliferating cells and is downregulated in tumor cells suggests that both ASncmtRNAs may act as tumor suppressors and might be involved in malignant cell transformation processes. Interestingly, SncmtRNA and ASncmtRNAs are not expressed in non-dividing cells. Therefore, the different expression profile observed allows to specifically distinguish between normal and tumoral cells [134,135]. The different expression of mitochondrial ncRNAs could be exploited in the oncology field both at a diagnostic [137] and therapeutic [138,139,140,141,142] levels. Consequently, in recent years several pre-clinical studies have been carried out. The studies conducted by Soledad and collaborators address this issue by demonstrating that the knockdown (KD) of ASncmtRNAs enhances apoptotic cell death in tumor cell lines without affecting the viability of normal cells [142]. In this study, KD of ASncmtRNAs was obtained through an antisense oligonucleotide (ASO), namely ASO-1537S, complementary to the loop region of both ASncmtRNAs. Various human tumor cell lines, such as HeLa, MCF7, and MDA-MB-231 (breast carcinoma), HepG2 (hepatocarcinoma), SiHa (cervix, HPV 16-transformed), DU145 and PC3 (prostate carcinoma), OVCAR-3, SKMEL-2 (melanoma), Caco-2 (colon carcinoma), A498 (renal carcinoma), U87 (glioblastoma), and H292 (lung carcinoma), were transfected with this ASO (Table 1). Using the TUNEL assay to evaluate DNA fragmentation and flow cytometric cell cycle (FACS) quantification to verify cell viability, the authors demonstrated that the construct induces cell death in all cell lines analyzed up to a level between 50% and 80% through the activation of apoptotic mechanisms. Particularly, it was demonstrated that transfection with ASO-1537S induced an inhibition of two members of the inhibitor in the apoptosis family of proteins (IAP) compared with control cells, i.e., Survivin and XIAP [142,143]. Survivin is involved in many cellular processes—such as the inhibition of autophagy and apoptosis, cell division—and promotes cancer metastasis and angiogenesis; it is often upregulated in many tumors [144]. XIAP also appears to be upregulated in many tumors and is involved not only in apoptosis but also in autophagy and necroptosis [143]. ASO-1537S induces Survivin downregulation in HnEMs (human normal epidermal melanocyte), PC3, SiHa, and H292 cells; the mechanism by which this regulation occurs involves Dicer (Table 1). In fact, the experimental results obtained suggest that the annealing of ASO with the loops of both ASncmtRNAs forms a double-stranded hybrid recognized by RNase H. The cleavage performed by RNase H would provide the substrate molecule processed by Dicer, which would then generate microRNAs capable of regulating Survivin [142].

Pre-clinical studies conducted by Lobos-González and coworkers in mice and murine cells have further confirmed the potential of ASncmtRNAs as therapeutic targets. In fact, in vitro KD of ASncmtRNAs with ASO-1560S reduces murine melanoma B16F10 cell proliferation through apoptosis (Table 1). Specifically, they observed a downregulation of Survivin in cells treated with ASO-1560S. Survivin mRNA levels are also lower than control, but less markedly than the protein decrease. Furthermore, treatment with ASO-1560S also induces a downregulation of the proteins N-cadherin (an epithelial–mesenchymal transition (EMT) marker), β-catenin, and a decrease in Rac-1 activity, thus suggesting a reduction in the metastatic invasiveness of the tumor. In vivo studies, performed by subcutaneous or intravenous inoculation of B16F10 melanoma tumors in C57BL/6 mice show results comparable to those described for B16F10 cell line. Indeed, they also observed both inhibition of tumor growth and metastasis formation to the lung and liver in mice treated with ASO-1560S [141].

Similarly, Borgna et al. analyzed the effect of the KD of ASncmtRNAs with ASO-1232S in vitro, using mouse renal adenocarcinoma cell line RenCa, and in vivo, using Balb/C mice inoculated with RenCa cells (Table 1). They found that KD induces cell death by apoptosis only in RenCa cell line but not in the control, consisting of normal epithelial cells freshly isolated from mouse kidney (mKEC). The analysis of the expression of the EMT markers, N-cadherin, and P-cadherin, shows a negative regulation of these factors suggesting that treatment with ASO inhibits the formation of metastases. Translation of these results to in vivo, using Balb/C mice inoculated with RenCa cells (subcutaneous, orthotopic and tail vein inoculation), shows that mice treated with ASO-1232S have a reduction in tumor growth and a decrease in the number of lung metastatic nodules. Molecular analysis of these same inoculated mice reveals the downregulation of both Survivin and matrix metalloproteinase 9 (MMP-9), both involved in the formation of metastases [140].

Recently, using cells derived from primary and metastatic clear cell renal cell carcinoma (ccRCC), the efficacy of treatment with ASO-1537 was tested both on primary cultures and on the orthotopic xenograft model of ccRCC [139] (Table 1). Using different experimental approaches such as Annexin V binding assay, pan-kinase inhibitor staurosporin, TUNEL assay, quantification by Western blot (WB) of Survivin protein levels, and PARP1 cleavage, the authors demonstrate that ASncmtRNA KD induces apoptotic death in primary ccRCC (PccRCC) cultures. The authors also observe that both the proliferative capacity and the ability to form metastases are reduced in cultured PccRCCs. At the molecular level, they explain the effect on proliferation with the reduction of cyclins B1 and D1 and the effect on metastasis with the reduction of N-cadherin. Instead, an in vivo model was obtained using small fragments of patient tumors implanted in immunocompromised mice (NOD/SCID mice). Both in vitro and in vivo experiments showed that the KD of both ASncmtRNAs with ASO-1537 causes an immediate arrest in cell proliferation followed by massive cell death. Using the orthotopic xenograft mouse model, the effectiveness of the therapeutic treatment was compared between ASO and Sunitinib, a molecule that constitutes a first-line treatment for ccRCC [147]. The results obtained show similar effectiveness between the two treatments [139]. However, while prolonged Sunitinib treatments can induce the development of resistance to the drug [148], for ASO, no cases of resistance have been reported thus far [149]. In the work of Varas-Godoy et al., the authors replace the ASO with an shRNA (short hairpin RNA). Lentiviral-encoded shRNA was used for the KD of ASncmatRNAs in vitro using cultured murine B16F10 and human A375 melanoma cells and in vivo using the B16F10 murine melanoma model (Table 1). Transduction of murine B16F10 and human A375 melanoma cells with the shRNA construct induces apoptosis in both cell lines. For in vivo analysis, C57BL6/J mice were injected subcutaneously with B16F10 tumor cells and then treated by intratumoral injection with shRNA. The obtained results showed a significant reduction in both tumor growth and the number and size of lung metastases compared to controls [138].

The processes that lead to the different expression of SncmatRNA and ASncmatRNAs in the above-mentioned cells and tissues are not yet fully understood. However, some studies conducted on human foreskin keratinocytes (HFK) immortalized with HPV demonstrate that the expression levels of the E2 protein, encoded by the HPV genome, are negatively correlated with the amount of ASncmtRNAs transcript. This correlation influences the extension of cellular replicative lifespan [150,151]. HPV is a family of viruses in which some subtypes are defined as “high-risk” because their prolonged infection, if untreated, can cause cancers of the cervix, anus, oropharynx, vagina, vulva, and penis. HPV subtypes 16 and 18 are the primary cause of 70% of cervical cancers in women [152]. The HPV genome encodes eight proteins; among these, E6 and E7 are considered oncoproteins capable of promoting carcinogenesis in infected cells, while E2 is described as a tumor suppressor gene capable of negatively regulating the E6 and E7 transcripts. Normal HFK expresses both SncmtRNA and ASncmtRNAs, but HFK cells immortalized with the complete genome either of HPV-16 (HFK698 cell line) or of HPV-18 (18Nco cell line) express only the SncmtRNA, ASncmtRNAs being downregulated. In both SiHa and HeLa cells, which arose from natural infection by HPV-16, and in cervical cancer cell lines, derived from infection by HPV-18, an expression of SncmtRNA and ASncmtRNAs similar to immortalized HFK cells is observed. Comparison of HFK698, 18Nco, SiHa, and HeLa cells with normal HFK suggests that ASncmtRNA-2 expression is downregulated during HPV-induced immortalization and before cellular transformation [151]. The comparison of the transcripts isolated between HFK cells transduced with a lentiviral construct HPV-18 encoding E2 protein and non-transfected control cells contributed to better clarifying the role of E2 in the regulation of both SncmtRNA and the two ASncmtRNAs [150]. The results obtained demonstrate that transduction with E2 induces an extension of replicative lifespan, from passage 8 to 26, correlated with E2 protein levels. Indeed, non-transduced cells enter senescence at passage 8, while cells transduced with HPV-E2 maintain their replicative potential until passage 26, which coincides with the decrease in E2 protein levels. After increasing E2 levels upon transduction, they found downregulation of ASncmtRNA-1 until passage 26. Instead, ASncmtRNA-2 decreases before transduction and then increases until passage 26. At passage 26, SncmtRNA also starts being downregulated in HFK-E2 cells. Instead, analysis of all transcripts (SncmtRNA and ASncmtRNA-1 and -2) in step 8 of non-transduced HFK cells show downregulation of SncmtRNA, upregulation of ASncmtRNA-2, and constant levels of ASncmtRNA-1. In addition to transcript levels, the mechanism of senescence was explored. FACS analysis of HFK-E2 cells at passage 26 shows arrest in the G2/M phase (presence of binucleated cells) as well as in the positive control consisting of HFK cells treated with colchicine. Instead, non-transduced HFK cells are arrested in the G1 phase. The activity of the cellular senescence marker SA-β-galactosidase was positive at passage 8 for HFK cells but negative until passage 26 for HFK-E2 cells. In summary, the downregulation of SncmtRNA and the upregulation of ASncmtRNA-2 suggest a pivotal role of these transcripts in replicative senescence (RS) [150].

The role in RS was also analyzed in the endothelial cells (ECs) that form the aorta of aged mice. In this system, ASncmtRNA-2 level is increased by approximately 3 times compared to ECs of young mice, and this increase correlates with the entry into senescence of ECs. Senescence is also confirmed by the upregulation of both p16 and p21 genes, typical RS markers, as they cause cell cycle arrest through the inhibition of cyclin-dependent kinases (CDKs). Finally, overexpression of ASncmtRNA-2 induces cell cycle arrest in the G2/M phase, reinforcing the idea that ASncmtRNA-2 regulates the cell cycle [153].

The molecular mechanism through which the KD of ASncmtRNAs induces cell cycle arrest, and therefore inhibits cell proliferation, is not yet fully clarified. However, the works of Fitzpatrick and Bendek show that the KD of ASncmtRNA influences the function of some key factors necessary for cell cycle progression [145,146]. Fitzpatrick et al. used the Andes-1537 ASO to perform ASncmtRNAs KD in three cell lines representing different subtypes of human breast cancer: MDA-MB-231 (triple-negative), MCF7 (ER-positive), and ZR-75-1 (HER2-positive) (Table 1). Consistent with previous works, the KD of ASncmtRNA influences the viability of all three cell lines not only by inducing cell death but also favoring a decrease of the proliferative index. In fact, the FACS analysis of cells transfected with Andes-1537 shows an increase in cells in S phase and a concomitant decrease in the G2/M population. Molecular analysis of factors involved in cell cycle regulation conducted on cells transfected with Andes-1537 shows the downregulation of cyclin B1, cyclin D1, CDK1, CDK4, and Survivin [145]. The data obtained for Survivin is consistent with what has already been illustrated above, where its function is associated with inhibition of apoptosis. However, in the context of cell division, Survivin is also involved in the mitotic spindle assembly checkpoint [154] and in the correct execution of cytokinesis [155,156], thus allowing progression through the M-phase of the cell cycle. Fitzpatrick and collaborators also demonstrate that KD of ASncmtRNA-2 with Andes-1537 induces the upregulation of mitochondrial encoded hsa-miR-4485-3p and hsa-miR-1973. To further investigate the role of these miRs, MDA-MB-231 cells were transfected with hsa-miR-4485-3p and hsa-miR-1973 mimics, finding that miR-4485-3p mimics induce downregulation of cyclins B1 and D1, while hsa-miR-1973 mimics apparently have no effect on cyclin regulation. Also, preliminary small RNA sequencing analysis identified several nuclear miRNAs upregulated in Andes-1537-treated MDA-MB-231 cells, such as hsa-miR-5096 and hsa-miR-3609. Using TargetScanHuman prediction, canonical binding sites of both miRs were identified on the CDK1 mRNA. The regulation of CDK1 by these miRNAs was confirmed using miRNA mimics: in MDA-MB-231 cells transfected with either mimic, a marked reduction of CDK1 occurs. To test the therapeutic potential of this antisense RNA, Balb/c mice were inoculated with MDA-MB-231 cells and subsequently treated with injections of Andes-1537. In this cancer xenograft model, a marked inhibition of tumor cell proliferation was observed [145]. Andes-1537 recently completed the Phase Ia Clinical Trial (NCT02508441), demonstrating not only that it is a well-tolerated drug, but also effective in patients with pancreatic cancer and cholangiocarcinoma, maintaining the disease stable beyond six months from the start of treatment [157].

Recently, the study by Bendek et al. [146] used an RNA sequencing and bioinformatic analysis approach to characterize differentially expressed cell cycle genes. The study was performed using the MDA-MB-231 cell line transfected with Andes-1537 (Table 1). Comparison between cells transfected with ASO and those not transfected (NT) or transfected with a control ASO (ASO-C) identified 168 downregulated genes and 164 upregulated genes in MDA-MB-231 cells transfected with Andes-1537 compared to controls. Functional classification showed that the upregulated genes mainly control cell cycle arrest and cellular response to DNA damage, while the downregulated ones are directly related to cell cycle progression. The authors further compared the gene expression profile determined in MDA-MB-231 cells transfected with Andes-1537 with additional cell lines, such as MCF7 and HMEC (normal human mammary epithelial cells), the latter used as controls. This comparison was aimed at identifying genes with similar expression profiles in different tumor cell lines to potentially recognize similar mechanisms of action. This selection allowed shrinking the list of possible candidates to three targets: CCNB1 (Cyclin B1), AURKA (Aurora kinase A), and TOPO2A (topoisomerase IIα). CCNB1 was chosen as an internal control since it has already been shown that the KD of ASncmtRNA in MDA-MB-231 induces downregulation of both mRNA and protein levels [145]. AURKA is a serine/threonine kinase involved in many processes carried out during cell division, such as centrosome duplication and maturation, G2/M transition, mitotic spindle assembly, and DNA replication [158]. In many solid tumors, AURKA overexpression correlates with genomic instability [159]. For this reason, AURKA is involved in numerous clinical trials aimed at identifying drugs that inhibit its activity [160,161]. Finally, TOP2A is an enzyme that participates in processes such as gene transcription, DNA replication, and cell division, where it plays a necessary role not only for mitotic chromosome condensation and segregation, but also for structural maintenance of chromosomes, thus ensuring genomic integrity. In fact, it has been observed that TOPO2A expression is altered in many tumor types [162] and its impairment leads to altered chromosome segregation and genomic instability [163,164]. TOPO2A is also an important therapeutic target of anticancer drugs; however, drugs currently in use are not specific enough and cause several important side effects, such as secondary cancers and cardiotoxicity [165]. Bioinformatic analysis of the data obtained from qRT-PCR and the subsequent WB analysis shows that ASncmtRNA KD induces a differential regulation of both AURKA and TOPO2A between the tumor cell lines MDA-MB-231/MCF7 and control cells HMEC. In fact, while AURKA mRNA levels decrease by approximately 2–3 times in all cell lines upon KD, AURKA protein levels behave differently: they decrease by approximately 6–8 times in tumor lines but increase by 11 times in HMEC cells. Instead, TOPO2A mRNA and protein decrease by approximately 2–3 times only in tumor lines, while the protein levels in the HMEC line are increased by 5 times (Table 2). The differential regulation observed between the two tumor lines and the control can be exploited for the development of new, more specific therapeutic strategies. For example, the use of ASO molecules that induce tumor-specific KD of ASncmtRNA could be used in combination with lower doses of traditional drugs. This approach would allow decreasing side effects resulting from current treatments and obtaining precision therapy [146].

The described function of ASncmatRNAs on the cell cycle appear to be mainly due to the altered expression of miRNAs and, among them, some studies have shown that ASncmatRNA-2 could act as their precursor [153,166]. Bianchessi and collaborators demonstrate, through bioinformatic analysis, that the sequence of the above-mentioned mitochondrial miRNAs, hsa-miR-4485 and hsa-miR-1973, is entirely contained in the long non-coding mitochondrial ASncmtRNA-2 [153]. To test the origin of these miRNAs, they used different experimental approaches. In cells expressing exogenous ASncmtRNA-2 treated with ethidium bromide (EtBr), which depletes the cell of mtDNA and precludes mitochondrial transcription, a downregulation not only of ASncmtRNA-2 but also of hsa-miR-4485 and hsa-miR-1973 was observed. Conversely, in EC cells transfected using a construct (pAS2) that contains the full-length sequence of human ASncmtRNA-2 under the constitutive promoter of the cytomegalovirus (CMV), the overexpression of exogenous ASncmtRNA-2 is observed, accompanied by a significant increase of only hsa-miR-4485. Analysis of the cell cycle in cells transfected with hsa-miR-4485 and hsa-miR-1973 mimics shows that the over-expression of the two miRNAs is correlated with a delay in G1 and G2/M phase progression. Finally, the authors used the microT-CDS bioinformatic tool to create a list of putative targets of hsa-miR-4485-3p and hsa-miR-1973, finding many cell cycle regulators. Although the authors suggest that the molecular mechanism underpinning cell cycle regulation deserves more in-depth studies, it would seem quite clear that both miRs are directly involved in its control [153]. In further support of the thesis that miR-4485 originates from ASncmtRNA-2, Farfan et al. demonstrated in vitro that Dicer co-immunoprecipitates with and processes ASncmtRNA-2, producing fragments corresponding to hsa-miR-4485-3p [166]. Finally, TaqMan quantitative assays performed on MDA-MB-231 cell line transfected with ASO Andes-1537 induced an increase of 6–7 times in the levels of both hsa-miR-4485-3p and hsa-miR-1973 compared to the control, probably with the involvement of Dicer [145].

### 5.2. LIPCAR

LIPCAR (long intergenic non-coding RNA predicting cardiac remodeling), is a chimeric mitochondrial transcript in which the 5′ half (1–384 nt) maps to the antisense strand of the mitochondrial lncCyt b gene and the 3′ half (385–781 nt) maps to the antisense strand of the mitochondrial COX2 gene [167]. LIPCAR was identified through a global transcriptomic analysis of patients with or without left ventricular remodeling after myocardial infarction [168]. Kumarswamy et al. demonstrated that LIPCAR is downregulated in the initial phase of myocardial infarction and upregulated in the later phases. Furthermore, LIPCAR upregulation is associated with increased cardiovascular death risk. These characteristics suggest its potential role as a biomarker of left ventricular remodeling and a predictor of patient survival upon heart failure [168]. Although the literature on LIPCAR is mainly focused on its role in heart disease, its involvement in cell proliferation and carcinogenesis has recently been explored. A model cell system used in these studies is human vascular smooth muscle cells (VSMCs). VSMCs are components of the aortic wall whose main function is to regulate vascular tension and maintain blood pressure. These cells are highly specialized and characterized by both a low proliferation rate and low protein synthesis activity. However, while both skeletal and cardiac muscle cells are terminally differentiated, VSMCs have reversible phenotypic plasticity in response to internal and external environmental stimuli [169,170]. When differentiated VSMCs perform the phenotypic switch, they undergo de-differentiation, which allows them to change their phenotype to a highly “synthetic phenotype”. Cells with the “synthetic phenotype” acquire various characteristics, such as proliferative and migratory capabilities, increased protein synthesis, and the secretion of large quantities of collagen, elastin, and matrix metalloproteinases. These characteristics are especially useful during tissue repair, but alterations in the switching process are also identified in many pathologies, including cancer [171]. In this context, alteration of the switching program is at the basis not only of the formation of both the pre-metastatic niche and of metastases [172,173] but also of vessel stability in tumors [174]. Therefore, these characteristics influence the malignant transformation by promoting phenomena such as invasion, metastasis, and tumor growth.

In 2019, Wang et al. analyzed the role of LIPCAR in the regulation of proliferation, migration, and change in the phenotype of VSMCs [175]. The authors measured LIPCAR expression levels using qRT-PCR in VSMCs treated with oxidatively modified low-density lipoprotein (ox-LDL) or platelet-derived growth factor BB (PDGF-BB). Both ox-LDL and PDGF-BB are key mediators of VSMC switching, promoting proliferation and migration in cultured VSMCs [176,177,178]. The results show that in cells treated with ox-LDL or PDGF-BB, LIPCAR levels increase compared to control and this increase correlates with the proliferation, migration, and phenotypic switching of VSMCs [175]. A similar result was obtained by transfecting cultured VSMC cells with a pcDNA-LIPCAR overexpression plasmid [175]. Specifically, in cells treated with ox-LDL or PDGF-BB, they analyzed cell proliferation rate using both the cell Counting Kit-8 (CCK-8) assay and densitometry analysis by WB of markers such as CDK2 and proliferating cell nuclear antigen (PCNA), which are upregulated, and p21, which is instead downregulated. The p21 protein functions as a cell cycle inhibitor and anti-proliferative effector in cells, therefore, its reduction is consistent with the proliferative state observed in VSMCs [179]. Instead, for cultured VSMCc transfected with an overexpression of pcDNA-LIPCAR, they studied their migration capacity by Transwell assay and by WB densitometric analysis of the markers MMP2 and MMP9. The results obtained highlight both a notable increase in cell migration and the upregulation of both MMP2 and MMP9. In addition, different markers of phenotypic switching, such as a-smooth muscle actin (a-SMA), tissue factor (TF), vascular endothelial growth factor A (VEGF-A), and angiopoietin 2 (Ang2), were analyzed as well. The contractile marker a-SMA, measured using immunofluorescence and WB assays, was lower than in the control, while the protein levels of VEGF-A and Ang-2, both detected by WB, were higher. Similarly, the ELISA assay showed an increase for TF [175].

In cancer, Bongolo and coworkers showed that LIPCAR is upregulated in both hepatocellular carcinoma (HCC) patients and HCC cell lines, compared to their respective controls [180]. In the first case, they analyzed circulating LIPCAR in the plasma of 64 patients with HCC using qRT-PCR, while in the second case, fluorescent in situ hybridization (FISH) was also conducted as a further test, in addition to qRT-PCR assay. To analyze the effect of LIPCAR upregulation, HepG2 cells were transfected with the PCDH-LIPCAR overexpression construct. Various tests were performed on the cells treated with PCDH-LIPCAR such as the TUNEL assay for the analysis of apoptosis, the CCK-8, which allows evaluating cell proliferation, and the Transwell and wound healing assays for measuring cell migration and invasiveness. The data obtained suggest that overexpression of LIPCAR induces an inhibition of apoptosis and an increase of proliferation, migration, and invasiveness of HepG2 cells. The mechanisms of migration and invasiveness were further investigated through WB by detecting the levels of the EMT markers E-cadherin, N-cadherin, Vimentin and Claudin. The results show an increase in the levels of N-cadherin, Vimentin and Claudin and a decrease in the level of E-cadherin [180]. The results obtained on HepG2 cells transfected with the PCDH-LIPCAR overexpression plasmid were then replicated in vivo. For this purpose, normal control cells, Hep3B cells, and Hep3B cells with LIPCAR overexpression were injected into mice and their livers monitored. Furthermore, to study metastasis, BALB/c nude mice were injected with HepG2 cells overexpressing LIPCAR, and both the liver and spleen were afterwards dissected and analyzed. The results suggest that overexpression of LIPCAR in mice induces HCC tumors and promotes the formation of metastases. At the molecular level, the expression of the proliferation markers Ki-67 and N-cadherin increased, whereas E-cadherin decreased. Overall, overexpression of LIPCAR promotes tumor growth and metastasis through activating the EMT process [180]. Although the mechanism underlying the multiple cellular events induced by LIPCAR deregulation remains largely undefined, the work by Wang et al. identified an interesting positive correlation between LIPCAR and the TGF-β/Smad pathway [181]. The authors investigated the potential role of the proliferation of atrial fibroblasts, induced by the upregulation of LIPCAR, in atrial fibrillation. The proliferation of atrial fibroblasts and the deposition of extracellular matrix (ECM) are related to the process of fibrosis, which influences the progression of atrial fibrillation [182]. Wang et al. found that LIPCAR and TGF-β1 were upregulated and positively correlated in atrial muscle tissues [181]. Peptide TGF-β1 (transforming growth factor-β1) is a multi-functional cytokine that activates the Smad pathway [183]. Wang et al. suggest that LICPAR regulates atrial fibrosis via modulating the TGF-β/Smad pathway [167]. The TGF-β/Smad pathway is involved in multiple cellular events, such as cell growth, differentiation, motility, and apoptosis [184]. Alterations in the regulation of this pathway have been associated with the development and progression of many tumors and with drug resistance processes [185,186,187,188,189,190,191,192]. It is therefore plausible to hypothesize that the elucidation of the mechanisms of action correlating LIPCAR to the TGF-β/Smad pathway may contribute, in the context of oncology, to the development of new therapies.

### 5.3. lncCyt b

In 2011, Rackham and collaborators identified three new lncRNAs transcribed from the human mitochondrial genome—lncND5, lncND6, and lncCyt b—through deep-sequencing data analysis and subsequent confirmation of expression by Northern blotting and strand-specific qRT–PCR [193].

Later, the work of Zhao et al. focused on comparing the localization of lncCyt b in HepG2 (hepatoma cells) and in HL7702 (normal hepatic cells). Using different experimental approaches, including FISH assay, MitoTracker staining and cellular fractionation assay, the authors observed that in HL7702 cells lncCyt b primarily localized in the mitochondria, whereas in HepG2 cells, it was still present in the mitochondria, but the most abundant pool was detected in the nucleus. LncCyt b levels were measured by real-time PCR using cDNA obtained from extracted and reverse-transcribed cytoplasmic and nuclear RNA. In addition, the possible relationship between lncCyt b and EMT was also analyzed. For this purpose, EMT was induced in HepG2 cells by stimulation with TGF-β1. Following stimulation, no changes in the nuclear localization of lncCyt b were observed in those cells. Overall, these results suggest aberrant shuttling of lncCyt b in the nucleus of liver tumor cells, where it may function in the epigenetic regulation of genes involved in tumor development/progression. However, further studies are needed to define the role that lncCyt b has within the nucleus and in carcinogenesis [194].

In the recent work by Zhang et al., a mechanism involving this lncRNA is described that, plausibly, could link it to carcinogenesis [195]. This work demonstrated the role of mitochondria-derived lncRNAs in heart failure. Using the cell lines AC16 (human cardiomyocytes), HL-1 (mouse cardiac muscle cells), and NMCM (neonatal mouse cardiomyocytes) as control cells, and a mouse animal model, it was proposed that cytosolic lncCyt b could function as a ceRNA to improve cardiac function in heart failure through the lncCyt b/miR-103-3p/PTEN/AKT axis. In particular, it was demonstrated that cardiac stress increased miR-103-3p levels by inhibiting the expression of lncCyt b, which caused a reduction of PTEN, leading to an enhancement of ROS formation and cardiac dysfunction [195]. Interestingly, the role of miR-103-3p in carcinogenesis is widely described in the scientific literature [196,197,198,199,200,201,202,203], and it was recently demonstrated that miR-103-3p/PTEN interaction regulates AKT by promoting both cell proliferation and cell invasion in non-small-cell lung cancer cells [204]. In addition, in some tumor types, it has been observed that miR-103-3p sponging by lncRNAs is involved in the regulation of different tumor hallmarks [205,206,207,208,209,210,211]. Thus, it is plausible to think that the lncCyt b/miR-103-3p/PTEN/AKT axis may be involved in carcinogenesis as well.

### 5.4. MDL1 and MDL1AS

Gao et al. used PacBio, a next generation sequencing (NGS) platform based on the single-molecule real-time (SMRT) sequencing technology, to analyze the transcriptome dataset of MCF-7 cell line to produce the full-length human mitochondrial transcriptome. In their study, the authors identified two new lncRNAs encoded in the mitochondrial D-loop region [212]. The two new lncRNAs were named Mitochondrial D-loop 1 (MDL1), which aligns on the sense strand of the D-loop, and Mitochondrial D-loop 1 antisense (MDL1AS), which aligns on the antisense strand of the D-loop.

Recently, Li et al. proposed that MDL1 is an RNA scaffold that tethers p53 and Tid1 (tumorous imaginal disc 1) together and has a role in the cell cycle through regulation of p53 activity [213]. Although the interaction between p53 and Tid1 has already been described in the literature [214], the novelty that emerges from this work is the formation of a ternary MDL1/p53/Tid1 complex that inhibits the nuclear translocation of p53 [213]. The protein p53 is a transcription factor with multiple targets—up to almost 350 genes are confirmed direct p53 targets, according to the study of Fisher’s [215]; these genes have roles in several cellular functions including apoptosis, cell cycle arrest, autophagy, metabolism, DNA repair, translational control, and feedback mechanisms. Consequently, the inhibition of p53 nuclear translocation has an ample regulatory effect on nuclear genes expression. The p53/MDL1 interaction was identified by Li and collaborators using an RNA library of A549 lung cancer cells on which they performed RNA-SELEX-seq (systematic evolution of RNA ligands by exponential enrichment followed by RNA sequencing). Then, to confirm the results, a native RNA immuno-precipitation (RIP) experiment was also performed. Experiments conducted on A549 cells overexpressing MDL1 and in p53-knockdown A549 cells suggest that p53 is necessary and sufficient for the function of MDL1. Indeed, in cells overexpressing MDL1, greater cell proliferation (CCK-8 assay), survival (colony formation assay), and migration (wound healing assay) is observed compared to the control, but these characteristics are suppressed in p53-knockdown A549 cells. Furthermore, by transfecting shRNA into A549 cells for the KD of either MDL1 or Tid1, the authors identified the genes specifically regulated by either protein. The analysis of only the genes co-regulated by MDL1 and Tid1, identified using the Kyoto Encyclopedia of Genes and Genomes (KEGG) pathway analysis, highlights an enrichment of genes involved in cell cycle control. Some of these genes, such as *p21*, *MDM2* (Murine double minute 2), and *CDK4* have been further validated both at the mRNA level, through RT-qPCR, and at the protein level, by immunoblotting. Finally, FACS analysis on A549 cells in which either MDL1 or Tid1 are knocked down, reveals the inhibition of the G1/S transition suggesting a synergistic interaction between Tid1 and MDL1 in controlling cell cycle. In conclusion, this work describes a mechanism through which MDL1 participates in retrograde signaling to mediate the regulatory control of nuclear genes involved in cell cycle regulation [213].

A recent work describes MDL1 and MDL1AS as prognostic biomarkers in rectal cancer and MDL1AS as a diagnostic biomarker for different types of cancer such as those affecting the colon, rectum, breast, and larynx [216]. The authors analyzed the expression levels of MDL1 and MDL1AS in tumor tissues and compared them to normal surrounding tissues. They found that MDL1 levels between the two samples are comparable, while MDL1AS levels are lower in rectal and colon cancers and higher in breast and laryngeal cancers. Then, they investigated the possible use of MDL1 and MDL1AS as prognostic biomarkers in rectal cancer. For this purpose, a clinical study was performed on patients with advanced rectal cancer (LARC) with a follow-up of more than 5 years, for which the possible correlation between the expression of MDL1/MDL1AS and LARC patient survival is investigated. Interestingly, the authors found a survival advantage for patients whose tumors show a higher level of expression of MDL1 or MDL1AS compared to patients with the same tumor type but with lower levels of expression for both MDL1 or MDL1AS. Although the result obtained for MDL1AS was more consistent, both markers, which show independent behavior between each other, could be used as prognostic biomarkers for colorectal cancer. To better understand the role of MDL1AS in tumors, double-stranded interfering RNAs (DsiRNAs) were used to downregulate MDL1AS in colon (HCT-116) and breast (MCF7 and MDA-MB-231) cancer cell lines. Using the Mito Stress Test on the SeaHorse platform, the effects of MDL1AS downregulation on mitochondrial respiration of HCT-116 and MDA-MB-231 cells were studied; MCF7 cells died after transfection with the DsiRNA. Results show that the downregulation of MDL1AS reduces several mitochondrial respiration parameters, such as basal respiration, maximal respiratory capacity, and ATP production. Instead, other parameters such as non-mitochondrial oxygen consumption, proton leak, spare respiratory capacity, or coupling efficiency do not show significant changes when compared to control. In general, the study of mitochondrial functionality shows similar behavior between the two cell lines, while the death of the third cell line remains unexplained. In addition to mitochondrial function, additional tumor hallmarks such as cell growth and migration were also analyzed. Comparison of the obtained results shows an opposite behavior between HCT-116 and MDA-MB-231 cells; in HCT-116 cells the downregulation of MDL1AS reduces both cell growth and migration, while the opposite behavior is observed in MDA-MB-231 cells. Finally, several markers of apoptosis (BAD, BAX, and BCL2) and cell cycle (CDK4, CDKN1A, and CCNA1) were analyzed. BCL2, CDK4, CDKN1A, and CCNA1 were all downregulated in both cell lines upon MDL1AS downregulation, compared to control cells not treated with DsiRNA. Instead, BAD and BAX showed downregulation only in HTC-116 and MDA-MB-231, respectively, when compared to control cells not treated with DsiRNA [216].

### 5.5. Circ-COX2

There are several publications describing the relationship between circular RNAs (circRNAs) and the progression of chronic lymphocytic leukemia (CLL), as well as the potential use of these molecules both as diagnostic biomarkers and as therapeutic targets [217,218,219]. Circ-COX2 is a new circRNA generated by back-splicing from the transcription of the mitochondrial COX2 gene. Also, the expression of this circRNA is related to the progression and prognosis of CLL, which is a form of B cell neoplasm [220].

Wu and collaborators focused on the potential role played by circ-COX2 in the progression and prognosis of CLL. In their work, they used different and complementary approaches to characterize plasma-derived exosomes of CLL patients [220]. Exosomes are small membranous vesicles involved in signal transduction and cell–cell communication; they are characterized by the presence of different types of molecules, including mRNA, ncRNA, and proteins. Interestingly, being embedded in plasma, they allow the exchange of information between cells either close to or distant from each other [221,222]. Additionally, circRNAs in exosomes have been described in certain cancers [223,224,225].

Wu et al. used electron microscopy, flow cytometry, WB of exosomal markers CD63, CD81 and TSG101 to confirm the purity of exosomes isolated from patients’ plasma. Then, by qRT-PCR they demonstrate that, in these patients, there is an exosomal enrichment of circ-COX2 compared to normal controls [220]. The functional analysis was conducted on three different CLL cell lines, namely MEC-1 (TP53 −/−), JVM-3 (TP53 +/+) and the primary cultures isolated from CLL patients (CLL-1). In all three cell lines, circ-COX2 was silenced by transfection with a siRNA (siRNA-circCOX2) [220]. Since TP53 is considered an important prognostic indicator in CLL [226] and is also involved in mitochondrial respiration [227,228], the authors looked for the possible relationship between circ-COX2 and TP53. Loss-of-function experiments performed on the aforementioned cells indicate that decreasing circ-COX2 is associated with the decrease of both mitochondrial membrane potential (measured by JC-1 fluorescent probe) and ATP production (ATP assay) [220]. The analysis of cell proliferation (CCK-8 assay) and apoptosis (FACS analysis) suggests that decreasing circ-COX2 both suppresses cell proliferation and increases apoptosis, when compared to cells transfected with negative controls. Overall, the authors demonstrate that circ-COX2 downregulation influences mitochondrial functionality, cell proliferation and apoptosis, while its upregulation is correlated with disease progression and a worsening survival [220]. Since both increased biogenesis and increased mitochondria number were observed in CLL [229], the authors investigated a possible positive correlation between the expression levels of circ-COX2 and the number of mitochondria. For this purpose, they analyzed the anti-leukemic activity of different compounds administered in MEC-1 and JVM-3 cells transfected in combination with siRNA-circCOX2. The compounds tested, using different concentrations, were as follows: carbonyl cyanide 3-chlorophenylhydrazone (CCCP), doxycycline, and metformin. CCCP leads to mitochondrial failure and cellular apoptosis [230,231], while doxycycline can disrupt mitochondrial biogenesis and is a cell growth inhibitor [232,233]. Metformin has many targets and is able to interfere with several cellular processes; it inhibits gluconeogenesis by acting on mitochondrial glycerol-3-phosphate dehydrogenase (mGPDH) and mitochondrial respiratory chain complex IV [234,235]; it promotes apoptosis by inducing the release of cytochrome c (Cytc) and increasing the expression of caspase-3 and caspase-9 [236]; a role for lysosomes was also revealed where it activates AMPK (AMP-activated protein kinase) [237]; it is an inhibitor of the mitochondrial respiratory chain [238]. The results show that the depletion of circ-COX2 by siRNA is sufficient to enhance the anti-leukemic activity of all tested compounds. The authors used four additional molecules (daporinad, PI-103, ABT-199 and OSI-027) administered individually in MEC-1 and JVM-3 cells in combination with siRNA-circ-COX2 to analyze a possible synergistic effect between the compounds and the silencing of circ-COX2 [220]. Daporinad, PI-103 and ABT-199 are usually used to induce apoptosis in CLL cells [239,240,241]. Instead, OSI-027 is a mTOR inhibitor [242] with a not yet characterized function in CLL. For all four compounds used in combination with siRNA-circ-COX2, they observed the inhibition of both circ-COX2 expression and cell proliferation. Furthermore, the effect of all four compounds was dose dependent and enhanced by silencing of circ-COX2 [220]. All together, these results suggest new therapeutic strategies for the treatment of CLL and the formulation of a different dosage of the drugs used with the aim of decreasing possible side effects of these pharmacological treatments.

### 5.6. mcPGK1

Chen et al., describe the role of mcPGK1 (mitochondrial circRNA for translocating phosphoglycerate kinase 1), a new circular RNA highly expressed in liver tumor-initiating cells (TICs) and in liver tumors [243]. TICs, also known as cancer stem cells (CSCs), represent a small population of cells with well-defined properties, such as self-renewal, differentiation, therapy resistance, and high tumorigenicity, which allow them to play a decisive role in the growth, heterogeneity, structure, recurrence, and metastasis of the tumor [244,245]. The authors, using primary liver cancer, separated liver TICs from non-TICs. Then, they isolated mitochondria from both cell types, and sequenced circRNAs. The comparison between the two samples (liver TICs/non-TICs) identified some circRNAs that are more expressed in liver TICs, including mcPGK1, which is transcribed from the CYTB gene locus (H strand of the mitochondrial genome). McPGK1 promotes a metabolic shift in TICs from OXPHOS to glycolysis (the previously described Warburg effect). The transition to glycolysis involves the action of the protein PGK1 (phosphoglycerate kinase 1), whose mitochondrial entry is favored by mcPGK1 [243]. Inside the mitochondrion, PGK1 phosphorylates PDK1 (Pyruvate Dehydrogenase Kinase 1), which, in turn, phosphorylates and inhibits the PDH complex. This molecular circuit inhibits OXPHOS and promotes glycolysis [246]. Glycolysis produces many metabolites, including α-ketoglutarate and lactate, which activate the Wnt/β-catenin pathway and this, in turn, promotes liver TIC self-renewal [243].

### 5.7. Circ-ND1 and Circ-ND5

Liu et al. identified hundreds of circular RNA molecules encoded by mitochondria and demonstrate that these have, at least in part, a role in promoting the entry of nuclear proteins into the mitochondria [247]. In particular, in their manuscript the authors show the expression levels of circ-ND1, transcribed from the mitochondrial ND1 gene, in 21 samples from patients affected by hepatocellular carcinoma (HCC) and compare them with normal adjacent cancer tissues as a control. The analysis suggests that in HCC, circ-ND1 is significantly upregulated. Although in smaller amounts, circ-ND5, transcribed from the mitochondrial ND5 gene, shows upregulation in HCC. However, the authors just report this observation, deferring any potential role of these circRNAs in the pathogenesis of HCC to future work.

## 6. Mitochondrial sncRNA

### 6.1. A Brief Overview of Validated vs. Non-Validated mtDNA-Encoded miRNAs

Several works are present in the literature that hypothesize the mitochondrial origin of specific miRNAs. Nonetheless, only miR-4485 [145,153,166] and miR-1974 [123] are presently considered ‘validated’ [248]. For miR-4485, we redirect the reader to paragraph 5.1 of this report, as it is an integral part of a lncRNA. As for the miRNAs for which a mitochondrial origin is hypothesized but not yet validated and which fit the scope of this review, i.e., they are involved in tumor development, growth, spread, or characterization, we included in this report the following: miR-1974, mir-1978 [123], miR-4461, mir-4463, and mir-4484 [113].

### 6.2. miR-1974

Bandiera et al. were the first to hypothesize the mitochondrial origin of miR-1974 by showing a perfect match between the miR-1974 sequence and the mitochondrial MT-TE gene, which encodes a tRNA for glutamic acid (E) [123]. In the same year, Ozata et al. describe its downregulation in adrenocortical carcinoma (ACC) [249]. Using qRT-PCR, the authors compared the expression levels of miR-1974 in ACC tumors (malignant), adenomas (benign tumors) and normal adrenal cortices, revealing a significantly lower expression of miR-1974 in carcinomas compared with adrenal cortices or adenomas. Later, Duregon et al. published a work that focused on the identification of dysregulated miRNAs in ACC (including miR-1974), which could be used as biomarkers of this tumor [250]. The results obtained demonstrate that the expression level of miR-1974 is associated with ACC tumors with low aggressiveness, low Ki-67 (used as a proliferation index), and a low ENSAT (European Network for the Study of Adrenal Tumors) stage. These characteristics, together with the not-appreciable expression difference between adenomas and carcinomas, challenge the use of this miR as a significant biomarker in adrenocortical tumors and does not allow it to be used as a biomarker in aggressive ACC.

### 6.3. miR-1978

Zhang et al. retrieved clinical data of patients with acute myeloid leukemia (AML) from the Cancer Genome Atlas (TCGA) database to study the miRNAs and mRNAs associated with this pathology. Using statistical methods (univariate Cox regression analysis) they looked for a correlation between the miRNAs and mRNAs associated with patients’ survival. Among the examined miRNAs, miR-1978 was considered a risk factor for survival time of AML patients [251]; however, the details of its role in AML remain to be explored.

### 6.4. miR-4461

Zhao et al. [252] analyzed the expression of miR-4461 in RCC samples and in different RCC cell lines comparing them with their respective controls. They also investigated the interaction, found with TargetScan, with the transcript of the 3C regulatory subunit of protein phosphatase 1 (PPP1R3C) using qRT-PCR and confirming the data using dual-luciferase reporter gene assay and RNA pull-down assay [253]. For the analysis of miR-4461 expression, the authors used six human RCC cell lines (SW839, A498, OSRC-2, 769-P, 786-O, and Caki-1), a human normal kidney cell line HK-2 as a control, patients’ RCC tissue samples, and adjacent non-carcinoma tissue as a control. Both in cells, particularly in the SW839 and OSRC-2 lines, and in RCC tissues, a reduction in the expression of miR-4461 was detected. The authors further analyzed the effect of miR-4461 on the phenotype of the SW839 and OSRC-2 lines. For this purpose, they transfected the two cell lines either with miR-4461 mimic or miR-4461 inhibitor and performed tests such as CCK-8 assay, BrdU ELISA assay, caspase-3 activity assay, and cell adhesion assay. The results of these tests suggest that in RCC cells, miR-4461 mimic induces a decrease in cell viability and proliferation, an increase in apoptosis, and a reduction in cell adhesion capacity. Since the effects of the miR-4461 inhibitor are opposite, they suggest that normal levels of miR-4461 suppress malignant phenotypes in RCC cells. Furthermore, the authors hypothesize that the suppression of the malignant phenotype is due to the inhibition of PPP1R3C. Indeed, in vitro studies using SW839 and OSRC-2 cells transfected with the miR-4461 inhibitor revealed that the decrease in miR-4461 expression correlates with the increase in PPP1R3C mRNA expression. At a phenotypic level, this correlation involves an increase in cell viability and proliferation, a decrease in apoptosis, and an increase in the capacity for cell adhesion. Overall, the results suggest that, in vitro, miR-4461 acts as an RCC inhibitor by targeting PPP1R3C. PPP1R3C is involved in glycogen biosynthesis and promotes both the accumulation and storage of glycogen [253,254]. Considering that deregulation of glucose metabolism is one of the tumor hallmarks [255] and that PPP1R3C is implicated in several cancers [256,257,258], understanding the mechanisms that regulate the PPP1R3C protein in RCC could be useful to better understand tumor progression and hypothesize potential therapies, using, i.e., miR-4461 as a possible PPP1R3R regulator.

Chen et al. [259] focused on the role of miR-4461 in exosomes derived from bone marrow mesenchymal stem cells (BMSCs) in the development of CRC. The authors initially demonstrated that the expression of miR-4461, both in the CRC cell lines (DLD1, HCT116, and SW480) and in the clinical tumor tissues of CRC, was lower than the corresponding controls, i.e., human normal colorectal mucosal cell FHC (fetal human cells) and normal tissues adjacent to the cancer. In addition, they also demonstrated that the expression of miR-4461 in BMSCs was higher than in CRC and FHC cell lines. Subsequently, using three prediction online tools (miRWalk, Targetscan, and miRDB) the authors hypothesized that the coatomer protein complex subunit beta 2 (COPB2) may be the target of miR-4461. This hypothesis was confirmed by a dual-luciferase reporter gene assay in CRC cells HCT116 and SW480. In addition, the study by qRT-PCR of the behavior of COPB2 mRNA in HCT116 and SW480 cells transfected either with miR-4461 mimic or with miR-4461 inhibitors showed that downregulation of miR-4461 induces an increase in COPB2 transcript, suggesting an inverse correlation between the two. Functional analysis of COPB2 conducted in HCT116 and SW480 cells treated with COPB2 siRNA or miR-4461 mimics also showed that the KD of COPB2 inhibits the proliferation, migration, and invasion abilities of CRC cells, suggesting that COPB2 behaves as an oncogene in this tumor. Finally, they also showed that BMSC-derived exosomes, containing high levels of miR-4461, inhibit COPB2 expression and the migration and invasion abilities of CRC.

Yan et al. [260] demonstrated miR-4461 downregulation in gallbladder cancer stem cells (CSC) and in GBC cell lines SGC-996 and GBC-SD and investigated its function in GBC cells as well as in NOD-SCID mice. SGC-996 and GBC-SD cells were transfected with constructs that overexpress or silence miR-4461. Results showed that the mRNAs of CSC markers, CD44 and CD133, were significantly decreased in cells overexpressing miR-4461, thus suggesting that miR-4461 inhibits gallbladder CSC expansion. NOD-SCID mice were inoculated with SGC-996 cells overexpressing miR-4461, showing a reduced tumor incidence compared to control. Furthermore, gain/loss of function experiments performed on SGC-996 and GBC-SD cell lines revealed that overexpression of miR-4461 inhibits cell proliferation and the ability to form metastases. This last aspect was also analyzed in BALB/c nude mice inoculated with SGC-996 miR-4461 mimic cells. In these mice, decreased numbers of pulmonary metastatic lesions were observed. Finally, the authors hypothesized that the mechanism of action of miR-4461 is due to its interaction with EGFR. In fact, a bioinformatics analysis revealed the presence in EGFR mRNA 3′-UTR of a potential binding site for miR-4461. Using different experimental approaches, including luciferase reporter assays, IHC staining, RT-PCR, and WB assay, the authors confirmed the bioinformatics prediction by also demonstrating the inverse relationship between mir-4461 and EGFR. Indeed, EGFR mRNA and protein expression were reduced not only in GBC cell lines overexpressing miR-4461 but also in mice inoculated with GBC cells overexpressing mir-4461. By transfecting SGC-996 and GBC-SD cells overexpressing miR-4461 with an EGFR siRNA, the authors also revealed that EGFR silencing abolished the self-renewal, metastasis and cell proliferation capacities of the treated lines compared to the control through the inactivation of the PI3K/AKT pathway. In fact, using cells overexpressing mir-4461, the reduction of both PI3K activity (kinase activity assays) and AKT phosphorylation levels (by WB assay) was demonstrated. As a proof of this, co-transfection in GBC cell lines with constructs overexpressing both mir-4461 and AKT recovers growth, self-renewal, and metastasis abilities of GBC cells.

Dou et al. [261] investigated the role of miR-4461 in HO8910 and A2780 human ovarian carcinoma cell lines, and in normal ovarian surface epithelial cells (OSE). They showed that in OC cell lines, miR-4461 deregulation, obtained by infecting the cells with miR-4461 sponge lentivirus and mimic lentivirus, deeply alters cell behavior: miR-4461 KD suppressed proliferation of OC cells, while miR-4461 mimic promoted proliferation of OC cells. These data were further validated through colony formation assay, 5-ethynyl-2′-deoxyuridine (EdU) staining, and flow cytometry; instead, miR-4461 KD did not affect the growth, viability and survival of OSE cells. Performing the wound healing assay showed that the miR-4461 KD ovarian cells had a poorer repairing ability, in contrast to the control group, and the transwell experiments highlighted an impaired ability of the treated cells to migrate and invade. Searching for a functional explanation of these behaviors, the authors found that miR-4461 has a potential binding site in PTEN mRNA 3′-UTR. To verify this possibility, luciferase activity assay was analyzed in wild-type and mutant PTEN 3′-UTR reporter plasmids that were transfected into miR-4461 KD or miR-4461 mimic cells, and their control ovarian cancer cells. In this way, they demonstrated that PTEN mRNA was indeed a direct target of miR-4461 in OC cells. These results were further validated using si-PTEN or control siRNA. Finally, the authors found that cisplatin resistance in OC cells is positively correlated to miR-4461 overexpression, while its KD causes cisplatin-induced growth suspension, making them more sensitive to this treatment. Altogether, these data indicate that miR-4461 is a potential onco-miRNA through targeting PTEN oncosuppressor and suggests possible ways to molecularly characterize and potentially treat specific types of OCs.

### 6.5. miR-4463

Modesto et al. [262] looked for the potential relationship between miRNA INDEL (insertions/deletions) mutations and susceptibility to gastric cancer (GC) in an Amazonian population. Starting from samples collected from both GC patients and healthy individuals, they used the Hardy–Weinberg equilibrium to find INDELs variants in genes encoding miRNAs expressed differently in GC samples compared to controls. Among the others, they identified miR-4463, for which they found significant associations of the Del allele variant, with an increased risk for the incidence of diffuse gastric adenocarcinoma. Furthermore, the analysis of the distribution of the Del miR-4463 variant as a function of age (less or more than 40 years) highlighted an association with a higher incidence in younger patients.

The work by Tan et al. [263] focused on the role of miR-4463 in CRC progression and metastasis. The authors, analyzing by qRT-PCR the non-metastasis and metastasis CRC tissues, and using adjacent normal tissues as controls, demonstrated that the expression of miR-4463 is increased in both non-metastatic and metastatic CRC tissue, although in the latter they have the highest levels. An increase in the expression of miR-4463 compared to the control (human normal colon epithelial cell line NCM460) was also demonstrated in three different CRC cell lines (HCT116, HT29, and LOVO). To further investigate the role of miR-4463 in CRC, the authors then analyzed LOVO cells, which represent the CRC cell line with higher expression of miR-4463. They transfected these cells with either miR-4463 mimic or miR-4463 inhibitor constructs to perform gain of function and loss of function experiments, respectively. Transfected cells were studied by MTT assay, colony formation assay, transwell assay, and flow cytometry assay. The results obtained showed that cells treated with miR-4463 inhibitor have a significantly decrease of cell viability, proliferation and migration, and an increase of apoptosis. Instead, cells treated with miR-4463 mimic showed opposite effects. To explain the mechanism of action of miR-4463, the authors used bioinformatics analysis to identify potential miRNA targets. From this analysis, they identified the best candidate as the Protein Phosphatase 1 Regulatory Subunit 12B (PPP1R12B) transcript. The miR-4463-PPP1R12B relationship was studied by dual-luciferase reporter and WB assays, which showed an increase in PPP1R12B protein in LOVO cells transfected with miR-4463 inhibitor, compared to control. Finally, co-transfection with miR-4463 mimic, which induces a decrease in mir-4463, and vectors overexpressing PPP1R12B, showed that overexpression of PPP1R12B recovers the effects of miR-4463 on viability, proliferation, and migration in LOVO cells. It is worth noticing here that PPP1R12B has phosphatase regulatory activity, and its dysfunction has been described in several tumors, such as esophageal cancer (EC) [264], CRC [265], and breast cancer (BrC) [266,267].

### 6.6. miR-4484

The analysis of the differential expression of miRNA in nipple discharge between benign tumors and BrC samples samples allowed the identification of several miRNAs that could be used as novel potential biomarkers for BrC. Among the those identified by qRT-PCR analysis, miR-4484 is present. This miRNA is upregulated in BrC and could be used as potential tumor biomarkers. However, the role played by miR-4484 in this condition remains to be clarified [268,269].

## 7. Conclusions

Non-coding RNAs represent most of the human genome activity. In the last few years, researchers have repeatedly shown that ncRNA are dysregulated in cancer cells, and in some cases, their molecular role has been reported in the control of gene and protein expression at transcriptional, post-transcriptional, and translational levels. This dysregulation can also be detected quantitatively (up- and/or downregulation of specific ncRNA), making these molecules powerful biomarkers not only for cancer detection but also in the molecular typing of tumors. Moreover, as detailed knowledge of their function improves, their possible role as molecular targets in precision medicine grows [270].

Mitochondria represent the “powerhouse of the cell” and, in normal tissues, their main roles are producing energy in the form of ATP/NADH and detoxifying ROS. In cancer, transformed cells rely much more on glycolysis than on mitochondria to produce energy; likely, this is due to the hypoxic microenvironment inside the tumoral mass coupled with the inefficient network of blood vessels formed during tumoral development. However, tumor cells cannot survive without them, as their complete depletion or metabolic inactivation causes cell death. This means that mitochondria still fulfill essential roles in cancer metabolism, but these roles only partially overlap those achieved in healthy cells. Unfortunately, several aspects of mitochondrial function in cancer are still far from being elucidated, but many laboratories are actively working to understand these roles.

The discovery that mtDNA also encodes ncRNA further complicates the scenario, introducing a direct role of these organelles in the epigenetic control of neoplastic transformation and tumor development. Although the study of mtDNA-encoded ncRNA is still in its infancy, it is very promising and likely will unveil some of the unknown roles mitochondria play in cancer. Moreover, their discovery further expands the list of possible anti-cancer targets, by means of developing and testing molecules that alter dysregulated ncRNA function. In this perspective, the study of mitochondrial ncRNA in the next few years will be central to cancer management.

## Figures and Tables

**Table 1:** Description of the main characteristics observed at the cellular and molecular level in different tumor cell lines or in animal models treated with ASO or shRNA for the knockdown (KD) of both ASncmtRNAs. The table summarizes the main characteristics of the ASncmtRNAs KD experiments described in detail in the text. KD experiments of ASncmtRNAs have been performed in different tumor cell lines or in murine animal models (column 2) using either antisense (ASO) or small hairpin (sh) RNA (column 1). In the first case, cultured tumor cells were transfected with ASO and shRNA constructs for the KD of ASncmtRNAs. The effect of silencing has been described at the cellular (column 3) and molecular (column 4) level. In vivo experiments were performed by inoculating tumor cells into mice to induce tumor formation. The animals were subsequently subjected to treatment with ASO. Tumor size and ability to form metastases were analyzed by comparing tumors from treated and untreated mice.

Silencing Strategy	Cell Line/Animal Model System ^1^	Cellular Effects	Molecular Target and Regulatory Effect	Ref.
ASO-1537	HeLa	Apoptosis induction. Cell proliferation and anchorage inhibition.	n/a	[142]
	SiHa	Cell death induction. Anchorage inhibition	Survivin downregulation	
	H292	Cell death induction	Survivin downregulation	
	SKMEL-2	Cell death induction. Anchorage inhibition	Survivin and XIAP downregulation	
	PC3	Cell death induction	Survivin and XIAP downregulation	
	OVCAR-3	Cell death induction. Anchorage inhibition	n/a	
	MCF7	Cell death induction	n/a	
	MDA-MB-231	Cell death induction	n/a	
	HepG2	Cell death induction	n/a	
	DU145	Cell death induction	n/a	
	Caco-2	Cell death induction	n/a	
	A498	Cell death induction	n/a	
	U87	Cell death induction	n/a	
ASO-1537	Primary and metastatic ccRCC cultures	Apoptosis induction. Cell proliferation and metastasis inhibition	n/a	[139]
	NOD/SCID mice inoculated with primary and metastatic ccRCC cancer cells	Tumor growth inhibition	n/a	
ASO-1537	MDA-MB-231	Apoptosis induction. Cell cycle arrest. Invasive capacity and stemness inhibition	CCNB1, CCND1, CDK1 CDK4 and Survivin downregulation hsa-miR-4485-3p, hsa-miR-4485-5p, hsa-miR-1973, hsa-miR-5096 and hsa-miR-3609 upregulation	[145]
	MCF7	Cell death induction. Invasive capacity and stemness inhibition	n/a	
	ZR-75-1	Cell death induction. Invasive capacity and stemness inhibition	n/a	
	Balb/c mice inoculated with MDA-MB-231 cells	Tumor growth inhibition	n/a	
ASO-1537	MDA-MB-231		AURKA, TOP2A downregulation	[146]
	MCF7		AURKA, TOP2A downregulation	
	HMEC		AURKA, TOP2A Upregulation	
ASO-1560	B16F10	Cell proliferation, stemness, invasiveness, anchorage and metastasis inhibition. Apoptosis induction	Survivin downregulation	[141]
	C57BL/6 mice inoculated with B16F10 melanoma cells	Tumor growth and metastasis inhibition	Survivin downregulation	
ASO-1232AS	RenCa	Cell proliferation, metastasis, anchorage, invasiveness inhibition. Apoptosis induction	Bcl-xL, Bcl-2, Survivin, N-cadherin and P-cadherin downregulation	[140]
	Balb/c mice inoculated with RenCa cells	Tumor growth and metastasis inhibition. Survival increased	Survivin and MMP-9 downregulation	
shRNA-Lv-sh-912 and shRNA-Lv-sh-1560	B16F10	Apoptosis induction	n/a	[138]
shRNA-Lv-sh-912	A375	Apoptosis induction	n/a	
shRNA-Lv-sh-1560	C57BL6/J mice inoculated with B16F10 cells	Tumor growth, number and size metastasis reduction	n/a	

^1^ Human tumor cell lines: HeLa, uterine cervical carcinoma; SiHa, cervix HPV 16-transformed; H292, lung carcinoma; SKMEL-2, melanoma; PC3 and DU145, prostate carcinoma; OVCAR-3, ovarian carcinoma; MDA-MB-231 (triple-negative), MCF7 (ER-positive), and ZR-75-1 (HER2-positive, breast carcinoma; HepG2, hepatocarcinoma; Caco-2, colon carcinoma; A498, renal carcinoma; U87, glioblastoma; ccRCC, clear cell renal cell carcinoma; HMEC, normal human mammary epithelial cells; A375, human melanoma. Murine tumor cell lines: B16F10, melanoma; RenCa, renal adenocarcinoma. n/a: data not available in the cited reference.

**Table 2:** Regulation of AURKA and TOPO2A upon ASO-1537-mediated KD of ASncmtRNAs, according to [146]. Quantitative changes in transcript (T) and protein (P) levels of AURKA and TOPO2a in MCF7, MDA-MB-231, HMEC cells after transfection with ASO-1537. The T and P levels of individual cell lines are compared to the same cell type not transfected with ASO-1537. MCF7 and MDA-MB-231, human breast carcinoma; HMEC, normal human mammary epithelial cells; AURKA, Aurora kinase A; TOPO2A, topoisomerase IIα; ↓, decrease; ↑, increase; NC, No Change.

Cell Line		AURKA	TOPO2A
MDA-MB231	T	↓	↓
	P	↓	↓
MCF-7	T	↓	↓
	P	↓	↓
HMEC	T	↓	NC
	P	↑	↑
